# St. John’s Wort for Depression: From Neurotransmitters to Membrane Plasticity

**DOI:** 10.3390/ijms262411925

**Published:** 2025-12-10

**Authors:** Verena M. Merk, Georg Boonen, Veronika Butterweck

**Affiliations:** Medical Department, Max Zeller Söhne AG, 8590 Romanshorn, Switzerland; verena.merk@zellerag.ch (V.M.M.); georg.boonen@zellerag.ch (G.B.)

**Keywords:** depression, St. John’s wort, Ze 117, lipidomics, membrane fluidity, *Hypericum perforatum*, neuroplasticity, phospholipids

## Abstract

Depression is a multifactorial disorder shaped by genetic, psychosocial, and biological influences, with hypotheses ranging from monoamine deficiency and neuroplasticity deficits to inflammation and stress-induced dysregulation. St. John’s wort (*Hypericum perforatum* L.) has long been used as an herbal antidepressant and is supported by clinical evidence for efficacy and safety in mild-to-moderate depression. While its multimodal mechanisms have been linked to neurotransmitter reuptake inhibition, neuroendocrine regulation, and modulation of neuroplasticity, recent findings suggest an additional role at the membrane level. Emerging lipidomic studies highlight that Ze 117, a low-hyperforin *H. perforatum* extract, counteracts stress- and glucocorticoid-induced increases in membrane fluidity by modulating lipid composition and cholesterol metabolism. These effects normalize receptor mobility and signal transduction, particularly of β1-adrenoceptors, and modulate glycerophospholipid metabolism in both cellular and animal models. Such membrane-stabilizing properties may represent a novel mechanistic pathway complementing classical neurochemical actions. This review revisits the mechanisms of St. John’s wort with a special focus on its impact on membrane lipids, positioning lipidomics as a promising tool for elucidating antidepressant activity. These insights may open avenues toward personalized therapeutic strategies in depression.

## 1. On the Origin of Depression: Integrating Psychosocial and Biological Perspectives

Depression is a multifaceted disorder, characterized by varying symptoms and degrees of severity. The identification of its underlying causes remains a significant challenge for clinicians and scientists.

Throughout history, a plethora of explanatory models have been proposed, encompassing psychosocial and biological aspects. While these models have each succeeded in capturing certain aspects of the disorder, they have frequently fallen short in addressing the comprehensive nature of the condition when considered in isolation. In recent decades, an integrative perspective has emerged, whereby psychological, social and biological factors interact in complex ways [1,2,3]. But there was a long way to go until then. One of the first psychosocial theories was established by the Austrian psychoanalyst Sigmund Freud in his essay “Mourning and Melancholia” (1917) [4]. Freud interpreted depression (or melancholia) as internalized feelings of self-blame and self-directed aggression arising, for example, in response to a conscious or unconscious significant loss, manifesting as feelings of guilt, low self-esteem, and sadness [4].

By the mid-20th century, cognitive theories shifted the focus toward conscious thought processes. For example, the American psychiatrist and psychotherapist Aaron Beck proposed that depression is based on a “negative bias” about the self and experiences while simultaneously blocking positive memories [5]. These schemata fuel cognitive distortions such as irrational beliefs, overgeneralization, and selective attention to negative information, which in turn maintain depressive symptoms [5]. Moreover, psychosocial theories explain depression within the context of social structures and individual life perspectives [6]. They assume that unstable social structures and environments, as well as destructive individual thought patterns, represent a significant risk factor for the development of depressive disorders [6].

Heritability studies have further shown an increase in depressive disorders within families, indicating a heritability estimate of approximately 37% for major depressive disorder [7]. This genetic component is part of the complex biological perspective on the development of depression. In addition to genetic factors, further biological hypotheses have emerged that could explain various aspects of the symptoms and the development of depressive disorders [2]. The initial hypothesis, designated as the “monoamine hypothesis”, emerged from the observation that various drugs that elevate monoamine levels alleviate depressive symptoms [8]. Based on the effects of tricyclic antidepressants (TCAs) and selective serotonin reuptake inhibitors (SSRIs), depression is interpreted as a neurotransmitter deficiency [8]. Although the effectiveness of these drugs is undeniable, the monoamine hypothesis alone is insufficient to provide a comprehensive explanation for depression [9]. This is evident from the fact that an increase in monoamine levels can be detected within hours after oral application, yet clinical improvement of symptoms only occurs after several weeks [10]. This prompted the development of hypotheses extending beyond a simple neurotransmitter deficit. A widely recognized hypothesis in this context is the “neuroplasticity hypothesis”. It associates depression with impaired synaptic plasticity and structural changes in the brain [11]. The delay in the onset of the clinical efficacy of antidepressants can be attributed to the time required to restore neuroplasticity through enhanced neurogenesis, synaptic remodeling and neuronal resilience [11]. Moreover, evidence implicates immune dysregulation, often referred to as “inflammation hypothesis”, as another key factor in the development of depression [12]. Elevated levels of pro-inflammatory cytokines (e.g., Interleukin-6 (IL-6), tumor necrosis factor (TNF), C-reactive protein (CRP)) are consistently observed in depressed patients and the experimental induction of inflammation triggers depressive symptoms [12,13]. Low-grade chronic inflammation may fuel pathways that can be linked to depressive symptoms, such as the body’s stress response [14,15]. The “stress hypothesis” proposes the correlation between hypercortisolemia and depression [2]. The mammalian stress response is regulated by the hypothalamus–pituitary–adrenal (HPA) axis [16]. Various stress signals stimulate the hypothalamus to produce and release corticotropin releasing hormone (CRH) which stimulates adrenocorticotropic hormone (ACTH) secretion from the pituitary gland [16]. ACTH reaches the adrenal gland through the circulation and induces glucocorticoid (cortisol, corticosterone) secretion from the adrenal cortex [16]. The system functions as a self-regulating control loop via feedback mechanisms [16]. This process is evolutionarily conserved and displays a central adaptation mechanism to maintain body homeostasis [16,17]. However, dysregulation leading to prolonged hypercortisolemia disrupts hippocampal homeostasis and impairs negative feedback loops, which may contribute to depressive episodes [18,19,20]. Furthermore, the impact of glucocorticoids on lipid membranes should not be disregarded in this context. Cortisol enhances membrane fluidity via non-genomic effects which affects receptor mobility, clustering and thereby signal transduction through membranes [21,22]. The downstream effects can be far-reaching, with the potential to influence the development and manifestation of depression [23]. In line with this, chronic stress and stress-induced oxidative processes can modify the lipidomic profile in the brain [24,25]. Moreover, a study on cortical cells has shown that cortisol induces neuronal depletion, which can be prevented by the presence of specific phospholipids [26]. Furthermore, chronic stress may alter membrane integrity by promoting the generation of reactive oxygen species (ROS) and reactive nitrogen species (RNS), which drive lipid peroxidation [27]. Oxidized lipids act as danger signals and have the capacity to amplify pro-inflammatory cytokine release [28]. This may result in a vicious cycle leading to disrupted membranes and inflammation.

Another intriguing hypothesis that links lipids and depression focuses on lipid rafts [29]. These cholesterol- and glycosphingolipid-rich microdomains are important for membrane receptor organization [29]. The integrity of lipid rafts appears to be crucial in the modulation of monoaminergic neurotransmission by influencing localization of receptors and adaptor proteins as well as downstream second messenger-mediated actions [29]. Alterations to lipid membranes may therefore provide a possible mechanistic link between external factors, metabolic disturbances and molecular dysfunction in depression.

However, to gain a comprehensive understanding of depressive disorders, an integrative approach is certainly the most promising option. Depression is influenced not only by inherited vulnerabilities but also by adverse environments, maladaptive cognition, and neurobiological dysregulation. Furthermore, a more profound comprehension of the mechanisms of action of antidepressants can also contribute to a more complete understanding of the development of depression. New findings on St. John’s wort, an herbal antidepressant that has been used for centuries, suggest that the impact on neuronal membranes may be more significant than previously understood.

## 2. St. John’s Wort: A Traditional Antidepressant—From Historical Use to Clinical Evidence

St. John’s wort (SJW, *Hypericum perforatum* L.), which is classified within the Hypericaceae family, is one of the most extensively researched medicinal plant. Its nomenclature reflects its flowering period around the feast of St. John the Baptist (24 June) and its leaves which seem perforated due to the presence of ethereal oil glands [30].

**Figure 1:**
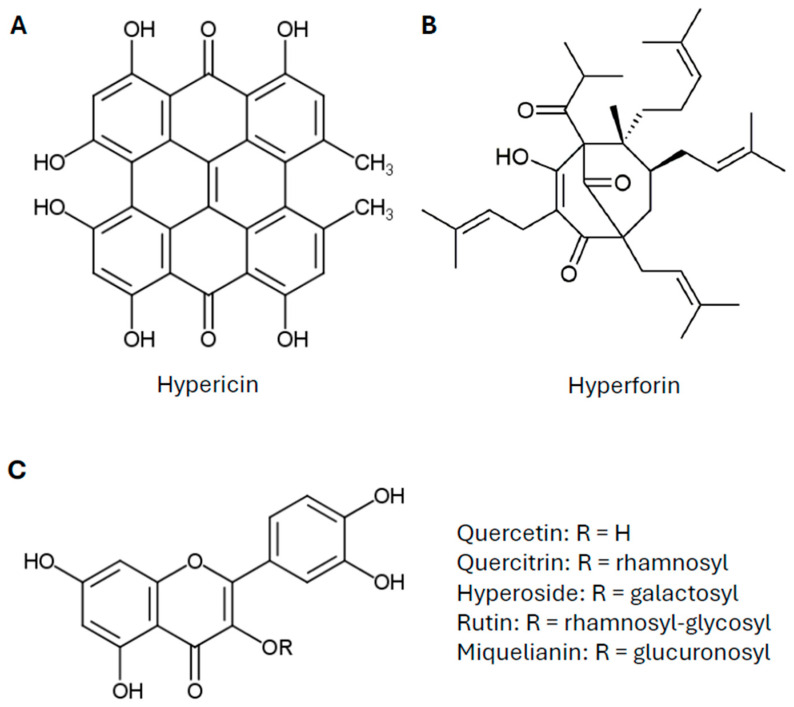
Chemical structures of the main ingredients of St. John’s wort. (**A**) Hypericin; (**B**) hyperforin; (**C**) flavonoid structure with respective residues.

Historical records document the use of SJW in early European herbal medicine. SJW has been employed in multiple forms, including oils, tinctures, and teas. The *Lorscher Arzneibuch* (8th century), one of the oldest European pharmacopeias, and Hildegard von Bingen (1098–1179), a German polymath, described the plant as a remedy for depressive states, melancholia, and nervous disorders [31,32]. Furthermore, the plant’s flowers were often macerated in olive or sunflower oil to produce a red-colored oil known as *Hypericum oil*, which was widely used both externally for the treatment of wounds, burns, and nerve-related pain, and internally for various ailments [30]. The main ingredients of SJW are considered to be hypericin, hyperforin and various flavonoids (Figure 1) [33].

By now, the use of SJW extracts in evidence-based phytomedicine is based on a plethora of clinical studies, overall supporting its beneficial effects in mild to moderate depressive states. A Cochrane review evaluating 29 trials that met the selection criteria came up with three main conclusions: (1) “[Hypericum extracts] are superior to placebo in patients with major depression”, (2) “[Hypericum extracts] are similarly effective as standard antidepressants” and (3) “[Hypericum extracts] have fewer side effects than standard antidepressants” [34]. The clinical studies on efficacy and safety of common Hypericum extracts such as WS 5572, LI 160, WS 5570 and Ze 117 have been extensively summarized before and are not within the scope of this review [35]. Despite its favorable safety profile, SJW is a prime example of pharmacokinetic interactions between herbal medicines and synthetic drugs. Not only professionals, but also laypeople are aware of the potentially weakening effect of SJW preparations on hormonal contraceptives, heart medications, and immunosuppressants. Reports of unwanted pregnancies and transplant organ rejections caused great uncertainty more than 20 years ago [36,37,38]. However, the mechanisms underlying these drug interactions are now well characterized, and with appropriate awareness, such interactions can be effectively prevented even in the context of polypharmacy [39].

**Figure 2:**
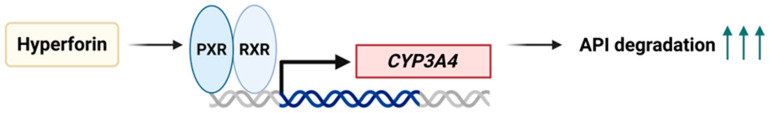
Mechanism of action of the pharmacokinetic drug interactions of St. John’s wort. Hyperforin, an ingredient of St. John’s wort, binds to and activates the nuclear receptor pregnane X receptor (PXR). Activated PXR forms a heterodimer with the retinoid X receptor (RXR) and binds to specific response elements in DNA, thereby inducing the expression of genes encoding cytochrome P450 (CYP450) enzymes (e.g., CYP3A4). The resulting upregulation of CYP450 enzymes enhances the metabolism and clearance of active pharmaceutical ingredients (APIs) that are substrates of this enzyme system, leading to reduced plasma concentrations and therapeutic efficacy of co-administered drugs. Created in BioRender.com. https://BioRender.com/qybq9to (accessed on 31 October 2025).

One of the most frequent causes of drug interaction is the influence on Cytochrome P450 (CYP450) enzymes in the liver [40]. Increased or decreased expression of these enzymes result in enhanced or reduced drug metabolism, respectively [40]. Consequently, this causes side effects with either reduced or enhanced efficiency [40]. In vitro analyses of isolated substances from SJW extracts were tested for their ability to activate pregnane-x-receptor (PXR), an important transcription factor that induce gene expression of CYP450 enzymes [41]. Within these studies, the phloroglucinol derivative hyperforin has been identified to be the only constituent that significantly induced PXR activity and thus CYP450 enzyme expression (Figure 2) [41]. The amount of hyperforin differs significantly in authorized SJW extracts, therefore several clinical studies investigated the drug-interactions induced by hyperforin-low and hyperforin-high extracts [39,42,43,44,45,46]. The pharmacokinetic drug interactions induced by hyperforin-low extracts were not clinically relevant and are therefore negligible [39,42,43,44]. This is of great importance especially for patients with multiple medications. Nevertheless, there is still an ongoing discussion about the importance of high hyperforin content for the antidepressant effect [33]. By now, it is evident that hyperforin alone is not responsible for the antidepressant effect and rather co-determinates the effect as part of the whole extract [33,47]. This is why the Herbal Medicinal Products Committee (HMPC) of the European Medical Agency (EMA) considers the whole extract as active ingredient and not a single constituent [47]. Nevertheless, the risk of pharmacodynamic drug interactions persists also with hyperforin-low extracts. In very rare cases, a combination of SJW and serotonin reuptake inhibitors or other serotonergic agents can result in the serotonin syndrome [46]. The excessive stimulation of serotonin receptors cause a range of various symptoms, including neuromuscular abnormalities, autonomic hyperactivity and mental impairments [48]. Consequently, it is critical to evaluate the risks and benefits when considering the concurrent administration of St. John’s wort and other medications.

## 3. Current Concepts in the Mechanisms of Antidepressants and St. John’s Wort

The mechanisms of action of antidepressants in general have been the focus of intensive research for decades, yet they remain incompletely understood. The earliest explanatory framework, the monoamine hypothesis, was derived from the observation that TCAs and SSRIs increase synaptic serotonin and noradrenaline within hours [10,49]. However, therapeutic benefits typically appear only after weeks, implying that acute monoamine elevation is not sufficient for clinical efficacy.

Contemporary concepts, as outlined by Cui et al. (2024), highlight antidepressants as modulators of a neurobiological network rather than single-target drugs [2]. Their effects span multiple, interconnected domains: neurotransmission, neuroplasticity, glutamatergic signaling, HPA axis regulation, immune and inflammatory pathways, mitochondrial metabolism, and circadian rhythm alignment. A unifying theme is the restoration of synaptic plasticity. Conventional antidepressants gradually enhance brain-derived neurotrophic factor (BDNF) signaling and tropomyosin receptor kinase B (TrkB) receptor activity, thereby promoting dendritic growth and resilience [50,51]. By contrast, rapid-acting agents such as ketamine block NMDA receptors on interneurons, trigger a glutamate surge, and activate mammalian target of rapamycin complex 1 (mTORC1)-dependent translation, leading to rapid synaptogenesis [50,52]. Antidepressants also normalize HPA axis hyperactivity, dampen pro-inflammatory cytokine responses, improve mitochondrial efficiency, and stabilize circadian rhythms. Taken together, these processes converge on restoring neuronal and network homeostasis, which appears to be the final common pathway of antidepressant efficacy.

Against this backdrop, the pharmacology of SJW illustrates the particular complexity of herbal medicines. Despite extensive research, no single constituent—whether hyperforin, hypericin, or flavonoids—can explain its clinical efficacy. As mentioned before, the whole extract is considered the pharmacologically active principle [53].

Pharmacological studies demonstrate that SJW inhibits the reuptake of serotonin, noradrenaline, dopamine, gamma-aminobutyric acid (GABA), and glutamate in neuronal preparations, thereby enhancing neurotransmitter availability [54,55,56,57,58,59]. These effects appear to result from indirect actions such as altered intracellular sodium or reserpine-like mechanisms, rather than high-affinity transporter binding [60,61]. In vivo, SJW extracts increase cortical monoamine concentrations [62]. In addition, receptor binding studies show low-affinity interactions with serotonin, noradrenaline, GABA, opioid, corticosteroid, NMDA, and neurokinin receptors [63,64,65,66,67,68,69,70,71]. Chronic treatment reliably downregulates β-adrenergic receptors, mirroring the adaptive receptor regulation seen with TCAs [72,73,74].

Beyond neurotransmission, SJW affects neuroplasticity, neuroendocrine function, and inflammation. Chronic administration alters hippocampal BDNF expression [75,76] and normalizes HPA axis hyperactivity, reducing CRH, ACTH, and corticosterone [77]. It also modulates FK506 binding protein 5 (FKBP5) expression, influencing glucocorticoid receptor sensitivity and stress resilience [78,79]. Anti-inflammatory properties are well documented, including reduced cytokine release in neuronal cells, protection against cytokine toxicity, and attenuation of systemic inflammatory responses [80,81,82,83].

Additional evidence comes from genomic and molecular studies. Jungke et al. (2010) showed that SJW and fluoxetine share the ability to reverse stress-induced gene expression changes in hippocampus and hypothalamus, regulating genes linked to oxidative stress defense, vesicle trafficking, and inflammatory signaling [84]. Interestingly, SJW induced broader transcriptional changes than fluoxetine and uniquely upregulated β-synuclein, a protein with neuroprotective properties. These data suggest that SJW, like SSRIs, targets fundamental stress-related molecular pathways while also exerting distinct neuroprotective effects.

In a recent study, El Hamdaoui et al. (2022) [85] identified transient receptor potential canonical 6 (TRPC6) channels as a molecular target of hyperforin. TRPC6-deficient mice displayed anxiety- and depression-like behaviors, and hyperforin was shown to activate TRPC6 selectively, enhancing hippocampal excitability and synaptic plasticity [85].

Consistent with these mechanistic insights, SJW extracts display robust antidepressant and anxiolytic activity in behavioral models such as the forced-swim test, tail-suspension test, learned helplessness, and cognition assays, with efficacy comparable to reference antidepressants [54,77,86,87,88,89,90,91,92,93,94,95,96,97,98,99,100].

**Figure 3:**
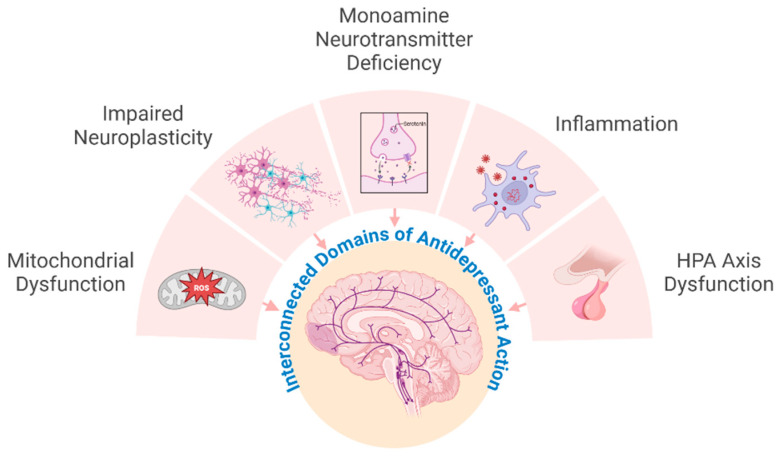
Interconnected domains of antidepressant action. Schematic representation of the major neurobiological domains implicated in antidepressant efficacy. Antidepressants modulate monoamine neurotransmitter signaling, enhance neuroplasticity, attenuate neuroinflammation, normalize hypothalamic–pituitary–adrenal (HPA) axis activity, and improve mitochondrial function. These domains are functionally interconnected, and disturbances in one system propagate across others, reflecting the network-based nature of depression pathophysiology and antidepressant mechanisms. Created in BioRender.com. https://BioRender.com/poizucx (accessed on 31 October 2025).

Taken together, SJW exerts its antidepressant activity through a multi-layered network of mechanisms, encompassing monoamine modulation, receptor adaptation, neuroplasticity, HPA axis normalization, immune regulation, and constituent-specific molecular effects such as TRPC6 activation (Figure 3). Yet, despite decades of research, these pathways still do not fully explain how a complex extract achieves clinically robust antidepressant effects. This gap has prompted increasing attention to the role of cellular membranes and brain lipids, which are central to receptor function, signal transduction, and stress adaptation. The next chapter will therefore focus on brain membrane lipids and explore how lipid remodeling may represent the missing link that integrates its diverse pharmacological actions into a coherent mechanism of antidepressant efficacy.

## 4. Beyond Synaptic Neurotransmission: The Role of Brain Lipids in Antidepressant Efficacy

The central nervous system (CNS) is unique among tissues in its exceptionally high lipid content. Nearly half of the dry weight of the brain consists of lipids, which form the structural matrix of neuronal membranes and provide the dynamic environment required for processes such as neurotransmitter release, receptor activation, and synaptic plasticity [101]. Membrane lipids not only determine the physical properties of neuronal membranes, such as viscosity, fluidity, curvature, and lateral heterogeneity, but also directly regulate the function of embedded proteins, including ion channels, receptors, and transporters [101,102]. Subtle changes in lipid composition therefore have profound consequences for neuronal excitability and signal transduction [101].

The lipid composition of neuronal membranes is highly complex, and each class of lipids contributes distinct physical and functional properties. Glycerophospholipids, which represent the majority of membrane lipids, are especially important for synaptic activity [103]. Their chemical diversity, reflected in the different headgroups such as phosphatidylcholine (PC), phosphatidylethanolamine (PE), and phosphatidylserine (PS), as well as in their acyl chain composition, controls membrane curvature, vesicle fusion dynamics, and the ability of proteins to interact with the bilayer [104]. Polyunsaturated fatty acids (PUFAs), such as docosahexaenoic acid (DHA) and arachidonic acid, are highly enriched in neuronal phospholipids. They confer high membrane fluidity and facilitate rapid synaptic vesicle turnover and receptor–effector coupling [101,105].

Cholesterol is another central regulator of neuronal membranes. It interacts with phospholipids to modulate bilayer order and to form specialized membrane microdomains, known as lipid rafts, which serve as organizing platforms for neurotransmitter receptors, G-proteins, and ion channels [106]. Adequate cholesterol levels are critical for neurotransmission and synaptic signaling, but imbalances of cholesterol content of membranes alter flexibility, lateral receptor distribution, and receptor mobility [107,108]. These processes can have direct consequences for signal transduction and synaptic plasticity, both of which are impaired in mood disorders as described before [2]. Moreover, cholesterol is the precursor of glucocorticoids. Elevated glucocorticoids, a hallmark of chronic stress, are believed to alter hippocampal structure and function with far-reaching consequences [25]. These findings may provide a mechanistic link between stress-induced hypercortisolemia and impaired neurotransmission.

Sphingolipids, including sphingomyelin, ceramides, and gangliosides, are highly enriched in neuronal membranes, particularly within lipid rafts [109,110]. Beyond their structural role, sphingolipids also function as bioactive signaling molecules. Ceramides, for example, are produced during cellular stress and modulate apoptotic pathways [111]. Chronic stress leads to elevated ceramide levels which have been consistently linked to neuroinflammation, impaired neurogenesis, and the development of depressive-like behavior in animal models, underscoring their pathogenic role in stress-related disorders [112,113,114]. Moreover, elevated ceramide levels have not only been detected in experimental stress models but also in the blood of patients suffering from depression, reinforcing their translational significance [115].

Plasmalogens, a subgroup of ether phospholipids, add another layer of complexity to neuronal membranes. These lipids are highly abundant in the brain and are characterized by their antioxidant capacity and their ability to influence vesicle fusion and membrane curvature [116]. Loss of plasmalogens, which occurs during aging and in neurodegenerative disorders, increases susceptibility to oxidative stress and compromises synaptic function [116].

This finely tuned lipid balance is highly vulnerable to environmental and biological stressors. Chronic stress and depression are increasingly recognized as conditions that profoundly alter lipid metabolism and membrane organization [25]. One of the most robust findings in clinical studies is a reduction in PUFA content, particularly DHA, in the brains and plasma membranes of patients with major depressive disorder [117]. Since DHA-rich phospholipids are essential for maintaining fluidity and synaptic adaptability, their depletion leads to stiffer membranes, diminished neurotransmitter release, and impaired plasticity [118]. This could display another important factor in the development of depression.

Finally, depression shares important features with the lipid changes observed in aging, as summarized by Skowrońska-Krawczyk et al. (2020). These include reduced levels of plasmalogens, shifts in the phosphatidylcholine-to-phosphatidylethanolamine ratio, and increased lipid peroxidation, all of which impair antioxidant defenses and weaken neuronal resilience [119]. Together, these processes render membranes more vulnerable to oxidative stress, which is heightened in both aging and depression, thereby compounding the vulnerability of neuronal networks [119].

In summary, chronic stress and depression produce a characteristic lipid signature consisting of reduced PUFAs, altered cholesterol distribution, elevated ceramide levels, and loss of protective plasmalogens. This constellation destabilizes receptor mobility, disrupts synaptic signaling, and perpetuates maladaptive stress responses through both neuroinflammatory and endocrine pathways. Lipids must therefore be considered not as passive scaffolds but as active regulators of neuronal function and plasticity. Their central role in synaptic physiology suggests that the membrane lipid environment may provide the missing link in antidepressant pharmacology. The following section will explore how St. John’s wort, particularly the low-hyperforin extract Ze 117, modulates neuronal membrane lipids and how such effects may complement classical neurotransmitter-based mechanisms to explain its clinical efficacy in depression.

## 5. St. John’s Wort and Membrane Plasticity: Bridging Classical Neurotransmission and Novel Mechanisms

A multifaceted mode of action of SJW is evident, and intensive preclinical research has now identified membrane lipids as a novel target of the hyperforin-low extract Ze 117 [120,121,122,123,124]. These findings suggest that the therapeutic potential of SJW extends beyond the modulation of neurotransmitters and receptors, into the fundamental biophysical properties of neuronal membranes.

The first evidence for membrane modulation by SJW extract came from experiments in rat glioblastoma (C6) cells exposed to cortisol, a glucocorticoid known to increase membrane fluidity. Using TMA-DPH fluorescence anisotropy, cortisol was shown to induce a dose-dependent increase in membrane fluidity. Chronic treatment with Ze 117, but not with citalopram and only weakly with desipramine, reversed this effect [120]. In addition, Ze 117 reduced the PC/PE ratio and inhibited the synthesis of saturated and monounsaturated fatty acids, thereby rigidifying the membrane. This normalization of membrane properties had functional consequences: the mobility of β1-adrenergic receptors, assessed by single-particle tracking of SNAP-labeled receptors, was reduced under Ze 117 treatment. Receptor trajectories could be categorized into three states according to diffusion speed: immobile (S1), slow-diffusing (S2), and fast-diffusing (S3). Ze 117 significantly increased the fraction of immobile receptors (S1) while reducing the slow-diffusing fraction (S2), leaving the fast-diffusing fraction unchanged [121]. Since lateral receptor mobility is crucial for efficient signaling but excessive fluidity impairs transmission, these data suggest that Ze 117 restores receptor dynamics to a physiological range, thereby optimizing signal transduction under stress conditions [125].

Similar effects were observed when membranes were challenged with dexamethasone. This synthetic glucocorticoid also increased membrane fluidity, an effect counterbalanced by Ze 117 [123]. At the molecular level, dexamethasone upregulated the expression of stearoyl-CoA desaturase 1 (SCD1), a rate-limiting enzyme in fatty acid desaturation, whereas Ze 117 downregulated its expression [123]. However, direct inhibition of SCD1 catalytic activity by Ze 117 could not be confirmed, as fatty acid desaturation ratios remained unchanged. Notably, Ze 117 reversed dexamethasone-induced fluidity changes to the same extent as the specific SCD1 inhibitor CAY10556 [123]. In addition, proteomic analyses revealed upregulation of multiple proteins involved in cholesterol biosynthesis following Ze 117 treatment, consistent with an observed increase in total membrane cholesterol [123]. Because cholesterol is a key determinant of membrane rigidity, low levels being associated with fluid membranes and high levels with rigid ones [126]. These findings suggest that Ze 117’s effects on fluidity are mediated, at least in part, by enhanced cholesterol synthesis. Hypericin may contribute to this process, since low concentrations of the pure compound were sufficient to increase cholesterol content in membranes [123]. Functionally, these changes translated into improved receptor signaling: Ze 117 rescued the dexamethasone-induced reduction in β-arrestin-2 recruitment following serotonin 1A (5-HT1A) receptor stimulation, indicating restored signal transduction capacity [123].

These findings were complemented by lipidomic analyses in human peripheral blood mononuclear cells (PBMCs) [122]. Cortisol treatment increased membrane fluidity and reduced overall lipid levels across most classes, with the exception of triacylglycerols (TAG) and cholesteryl esters (CE), which remained unchanged. Co-treatment with Ze 117 counteracted these effects, tending to normalize lipid profiles and significantly upregulating TAG and CE. Detailed analysis revealed that cortisol increased chain length and unsaturation of phosphatidylcholine species, whereas Ze 117 reversed these changes [122]. In PE and phosphatidylcholine ethers (PC-O), Ze 117 reduced chain length and double bonds, though the physiological significance of these changes remains to be fully clarified. Overall, the data indicate that the primary drivers of Ze 117’s effects on fluidity are changes in cholesterol metabolism and modulation of double bond content in phospholipids [122,123].

Evidence from animal models further supports these cellular findings. In rats exposed to chronic corticosterone, Ze 117 reduced immobility in the forced swim test, confirming its antidepressant-like activity [124]. Plasma lipidomics revealed that corticosterone increased the average number of double bonds in plasma glycerophospholipids, while Ze 117 co-treatment reduced this effect to control levels. Interestingly, similar results were observed with escitalopram, suggesting a shared impact of antidepressants on glycerophospholipid metabolism [124]. In hippocampal samples, Ze 117 and escitalopram increased levels of lysophosphatidylethanolamines (LPEs), and higher LPE content positively correlated with improved behavioral performance in the forced swim test, specifically with longer latency to immobility [124]. This indicates that modulation of glycerophospholipid metabolism may contribute directly to the behavioral efficacy of antidepressants.

Although cell culture systems and plasma levels do probably not reflect the situation in the brain directly, they are indispensable for mechanism of action studies due to their availability. Blood is expected to be an indicator for changes that take place in other organs, including the brain [127]. Given the unavailability of human brain samples from patients suffering from major depressive disorder (MDD) due to ethical considerations, research cannot focus solely on brain samples in animal models but must also examine blood samples. Moreover, it is currently unknown which brain regions are affected by lipidomic changes, and whether there are individual differences in these regions depending on the severity of MDD. Consequently, until further biologically and translationally relevant methods are developed, blood samples remain the best option.

**Figure 4:**
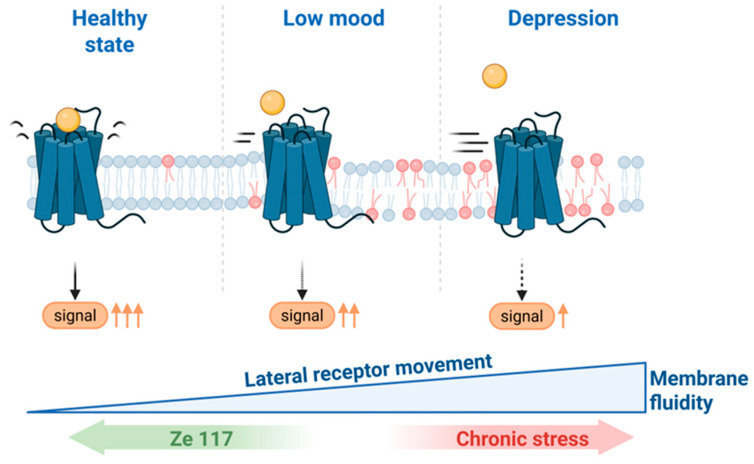
Hypothesized mode of action of Ze 117. The membrane properties of depressed individuals differ from those of healthy individuals. However, the transitions are not clearly defined. Chronic stress leads to persistently high cortisol levels in the blood which are believed to be associated with depression. Cortisol has been demonstrated to alter cellular membranes by making them more fluid and consequently modifying the lateral mobility of receptors such as β1-adrenergic receptors. Consequently, signal transduction becomes restricted. Ze 117 may normalizes signal transduction by reversing cortisol-dependent changes in membrane fluidity. Such changes may be indicated by the amount of double bonds and the chain length of membrane lipids. This finding provides a partial explanation for the antidepressant properties of Ze 117. Created in BioRender.com. https://BioRender.com/y14nkjc (accessed on 31 October 2025).

Together, these studies highlight a novel mode of action of SJW that extends beyond the classical neurochemical framework. Chronic stress and elevated glucocorticoids induce pathological changes in membrane fluidity, lipid composition, and receptor signaling. Ze 117 reverses these changes by restoring lipid balance, enhancing cholesterol content, adjusting fatty acid composition, and normalizing receptor mobility. The net result is stabilization of membrane properties and restoration of efficient signal transduction through receptors such as β1-adrenergic and 5-HT1A receptors (Figure 4).

## 6. Conclusions and Future Perspectives

The emerging evidence on Ze 117 demonstrates that SJW acts not only through modulation of neurotransmitter systems, receptor interactions, and HPA axis regulation but also by stabilizing the lipid environment of neuronal membranes. By restoring membrane fluidity, normalizing receptor mobility, and influencing cholesterol and phospholipid composition, Ze 117 supports efficient serotonergic and adrenergic signaling. This lipid-centered mechanism thus bridges classical neurochemical pharmacology with a broader systems-level perspective, positioning membrane lipids as the missing link that integrates SJW’s multifaceted pharmacology.

Beyond explaining clinical efficacy, these lipid effects open new avenues for biomarker discovery and therapeutic innovation. Lipidomic signatures, such as changes in PCs, LPEs, or cholesterol-associated profiles, may possibly serve as translational biomarkers to monitor treatment response and stress adaptation. The identification of specific lipid species as biomarkers may also facilitate the diagnosis of early-onset depression in certain cases. Furthermore, these biomarkers could guide individualized treatment strategies, potentially in an algorithmic manner, paving the way toward personalized medicine in psychiatry. However, the prediction and actual verification of human membrane lipids as biomarkers with actual clinical utility will require human clinical studies because previous hints towards lipid biomarkers in depression are mostly based on in vitro and rodent studies.

Taken together, membrane lipids emerge not only as passive structural components but as dynamic therapeutic interfaces. Their modulation by SJW extracts such as Ze 117 provides a unifying framework for its antidepressant action and highlights novel opportunities for precision medicine in depression. Nevertheless, further research, particularly clinical studies, is required to verify these findings and to translate lipid-based mechanisms and biomarkers into therapeutic practice. Importantly, the current evidence derives from studies with the low-hyperforin extract Ze 117, and it remains to be established whether high-hyperforin extracts exert comparable effects on membrane lipids or whether their pharmacology differs in this respect.

## Data Availability

No new data were created or analyzed in this study. Data sharing is not applicable to this article.

## Abbreviations

The following abbreviations are used in this manuscript:

5-HT1A	Serotonin 1A receptor
ACTH	Adrenocorticotropic hormone
BDNF	Brain-derived neurotrophic factor
CE	Cholesteryl esters
CNS	Central nervous system
CRH	Corticotropin releasing hormone
CRP	C-reactive protein
CYP450	Cytochrome P450
DHA	Docosahexaenoic acid
EMA	European Medical Agency
FKBP5	FK506 binding protein 5
GABA	Gamma-aminobutyric acid
HMPC	Herbal Medicinal Products Committee
HPA	Hypothalamus–pituitary–adrenal axis
IL-6	Interleukin-6
LPEs	Lysophosphatidylethanolamines
mTORC1	Mammalian target of rapamycin complex 1
PBMCs	Peripheral blood mononuclear cells
PC	Phosphatidylcholine
PC-O	Phosphatidylcholine ethers
PE	Phosphatidylethanolamine
PS	Phosphatidylserine
PUFAs	Polyunsaturated fatty acids
PXR	Pregnane-x-receptor
RNS	Reactive nitrogen species
ROS	Reactive oxygen species
SCD1	Stearoyl-CoA desaturase 1
SJW	St John’s wort
SSRI	Selective serotonin reuptake inhibitors
TAG	Triacylglycerols
TCAs	Tricyclic antidepressants
TNF	Tumor necrosis factor
TrkB	Tropomyosin receptor kinase B
TRPC6	Transient receptor potential canonical 6
